# Specific lumbar puncture training during clinical clerkship durably increases atraumatic needle use

**DOI:** 10.1371/journal.pone.0218004

**Published:** 2019-06-10

**Authors:** Xavier Moisset, Bruno Pereira, Carole Jamet, Alexandre Saturnin, Pierre Clavelou

**Affiliations:** 1 Service de Neurologie, CHU de Clermont-Ferrand, Université Clermont Auvergne, Clermont-Ferrand, France; 2 Université Clermont Auvergne, Inserm Neuro-Dol, Clermont-Ferrand, France; 3 Biostatistics Unit, DRCI, CHU de Clermont-Ferrand, Clermont-Ferrand, France; Taipei Veterans General Hospital, TAIWAN

## Abstract

**Background:**

Atraumatic needles are proposed to lower complication rates after lumbar puncture (LP). Only a minority of physicians use such needles. Here we aimed to assess the impact of specific training in LP during clinical clerkship on the proportion of medical students using atraumatic needles.

**Methods:**

We performed a case-control study comparing medical students undergoing clinical clerkship and students undergoing specific LP training. The 176 students of a class underwent training in LP just before beginning their clinical rotations. This training consisted of 45 minutes of theoretical training and a 90-minute practical session with a dummy. Twenty students were selected from the class at random, and their competence was assessed with a multiple choice questionnaire (MCQ) and an objective structured clinical examination (OSCE), nine months after the specific training. These 20 cases were compared with 20 students randomly selected from a class of 180 students who had not undergone specific training in LP and were at the end of their clinical clerkship.

**Results:**

We found that 60% of the students with specific training and 25% of those with classic clinical training used an atraumatic needle during the OSCE (*p* = 0.025). The mean MCQ (/100) scores obtained were 57±15 and 60±15 for the specific and classic training groups, respectively (*p* = 0.35). Overall OSCE score was similar in the two groups (63.5±9.3 vs. 65.8±9.3; *p* = 0.20).

**Conclusion:**

Very few practicing physicians use atraumatic needles, which limits the teaching of their use to medical students. Specific training durably increases the use of appropriate needles.

## Introduction

**Fig 1:**
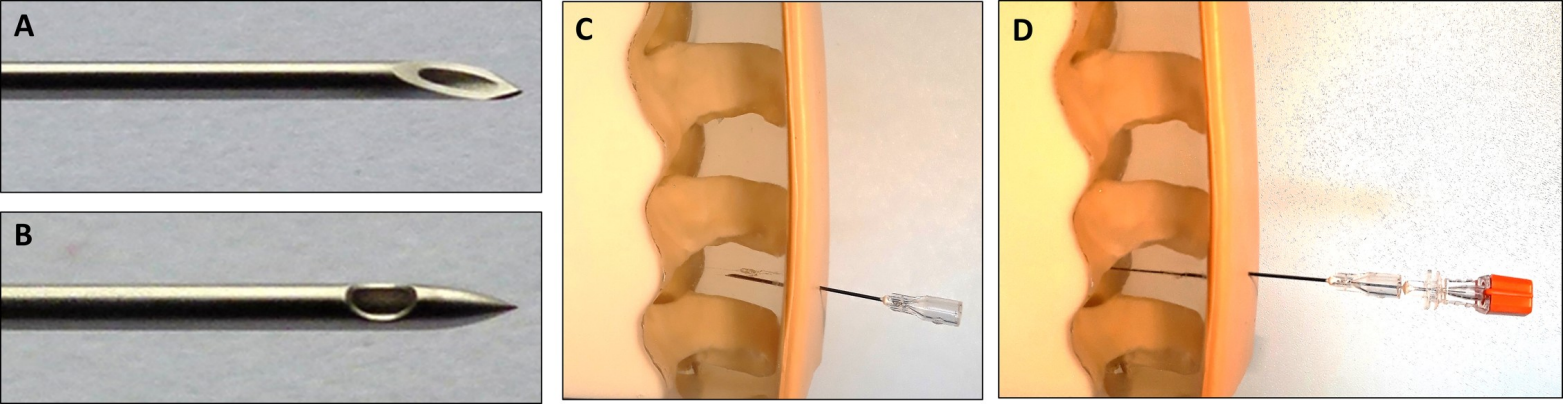
Illustration of atraumatic and cutting lumbar puncture needles. A: Pencil point (atraumatic) lumbar puncture needle. B: Beveled (cutting) lumbar puncture needle. C: Introducer needle to go through the skin and inter-spinous ligament. D: Pencil point (atraumatic) lumbar puncture needle to be inserted in the introducer and go through the dura mater.

Pencil point (atraumatic) needles (Fig 1) are recommended over beveled (cutting) needles for lumbar puncture (LP), to prevent complications relating to this procedure and reduce costs to the healthcare system [1]. This is well-known for more than 20 years, especially among neurologists [2,3]. Neurologists are the principal group of doctors performing LP. Nevertheless, many LPs are also performed by other hospital physicians [4]. Very few practicing physicians use atraumatic needles [4,5], which limits the teaching of the use of these needles to medical students.

In France, medical students undergo three years of preclinical and three years of clinical training. They generally observe and practice LP directly on live patients during their clinical rotations. Atraumatic needles have been used since 2010 in the neurology department of Clermont-Ferrand University Hospital. Nevertheless, less than 10% of the LPs performed at this hospital in 2014 involved the use of such needles, indicating that practices are changing slowly, if at all, in the hospital as a whole [4]. A specific training course for medical students beginning their clinical clerkship was implemented in September 2016, to try to accelerate this change.

The primary outcome of this study was the evaluation, with an objective structured clinical examination (OSCE), of the proportion of medical students using an atraumatic or cutting needle as a function of the type of training they had received (classic training during clinical rotations only or specific training before the first clinical rotation). The secondary outcomes concerned the students’ theoretical knowledge and ability to perform LP. Satisfaction was also evaluated for the students undergoing specific training.

## Methods

### Subjects

In September 2017, all the medical students (*n* = 176) beginning their 4th year in the medical school (1st year of clinical clerkship) received specific training in LP before the first clinical rotation.

The control group consisted of 6th year medical students (*n* = 180). These students had gone through the entire three years of classic clinical clerkship. Fifteen emergency medicine residents and fellows were also evaluated before receiving a specific training for atraumatic LP needle use.

For studies concerning medical education (only medical students involved and not patients) ethical review board is not applicable. Verbal informed consent was obtained from each participant.

### Specific training

For the specific training, the students attended a 45-minute lecture and underwent 90 minutes of practical training on LP simulator (Lumbar Kun II, Kyoto Kagaku, Kyoto, Japan). Preparation of this type has already been reported to be appreciated by medical students[6]. During the practical training, the students had to learn how to place a patient comfortably in a sitting or lateral decubitus position. They had to obtain artificial CSF with both 22G cutting needles and 25G atraumatic needles. During the entire training program, they were supervised by specifically trained 5^th^ year students and by a fellow in neurology (XM). The students’ satisfaction with this training was evaluated on a five-point Likert scale.

### Evaluation of practical skills

Twenty students at the end of the 4th year and 20 at the end of the 6th year of medical training were randomly selected for this evaluation. Each of these students was presented with the same standardized scenario. The student was asked to manage a 23-year-old woman attending the emergency department for an unusual headache of 24 hours’ duration associated with fever (38.5°C). The patient had no significant medical history. Physical examination revealed a meningeal syndrome. There was no purpura or hemodynamic instability. Two evaluators were present: the simulated patient (CJ) and a simulated nurse (XM), who asked the student to obtain a blood sample or assist with LP. A 21-item evaluation chart was used (S1 Appendix). The evaluation had to be completed in 20 minutes. The dummies used for the evaluation were identical to those used for the practical training.

### Evaluation of theoretical knowledge

A 10-question multiple-choice questionnaire, with five possible answers to each question was used to assess the knowledge of the students (S2 Appendix). Fourth year medical students were evaluated just before and just after the specific training, and then nine months later. The control group was evaluated only once, at the end of the 6^th^ year of medical training. This questionnaire assessed the students’ basic knowledge about anatomy, to the risks of LP and the interpretation of results. The questionnaire was also completed by 15 emergency medicine residents and fellows.

### Statistics

Qualitative variables are expressed as percentages. Quantitative variables are presented as means and standard deviations if normally distributed, or as medians and interquartile ranges if not. The normality of the distributions of variables was assessed with the Shapiro-Wilk test. For qualitative variables, the significance of differences was assessed with Pearson’s chi^2^ test or Fisher’s exact test, as appropriate. For quantitative variables, we used Student’s *t*-test or Welch’s test, as appropriate (taking the heterogeneity of variances into account). For comparison concerning 3 groups, ANOVA was conducted. For the evolution of scores, an ANOVA for repeated measures was performed, applying Sidak correction to take multiple comparisons into account. All statistical tests were two-tailed, and values of *p*<0.05 were considered significant.

## Results

Both theoretical and practical skills were evaluated in June and July 2018, nine months after the specific LP training for 4^th^ year students and at the end of the 6^th^ year of medical studies for the control group (raw data are available in the S3 Appendix).

### Evaluation of practical skills

**Fig 2:**
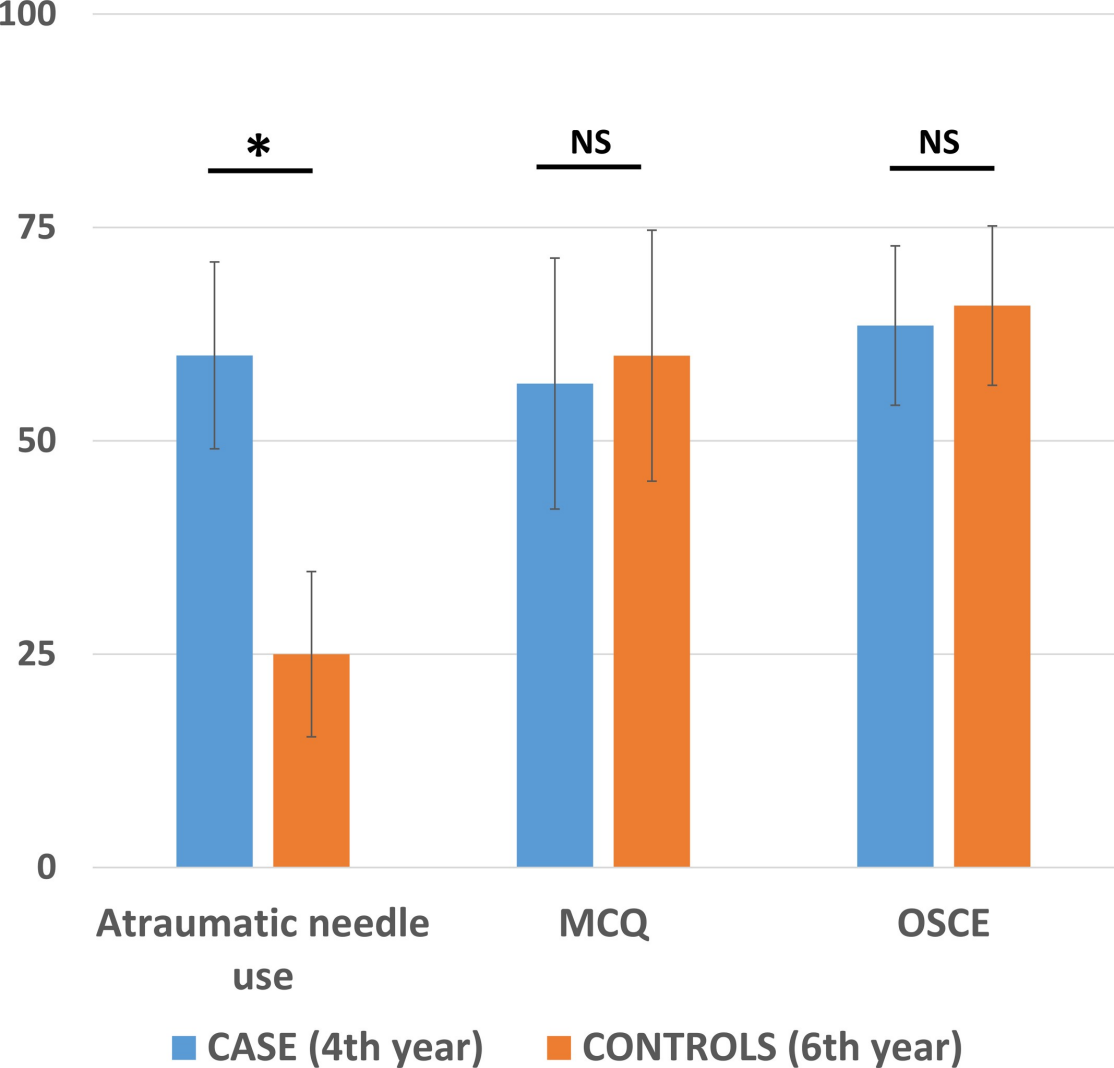
Proportion of students using an atraumatic needle during the evaluation, theoretical and practical scores. Percentage, multiple choice questionnaire (MCQ) and objective structured clinical examination (OSCE) scores for 4th year students with specific training and for 6th year students after classical clerkship are presented. Scores are expressed out of 100 and the standard deviation is shown. Atraumatic needle use differed significantly between the two groups of students (*p* = 0.025). NS: no significant difference.

Atraumatic needles were used for LP by 60% of the 4th year students who had received specific training, and 25% of the students after classic clinical clerkship (*p* = 0.025; Fig 2). None of the emergency medicine residents and fellows were using atraumatic needles in their daily practice at the time of evaluation. The OSCE scores obtained were 63.5 ± 9.3 for case students and 65.8 ± 9.3 for the control group (*p* = 0.20; Fig 2). Overall, 50% of cases and 65% of controls were able to obtain artificial CSF from the simulator (*p* = 0.34).

### Evaluation of theoretical knowledge

**Fig 3:**
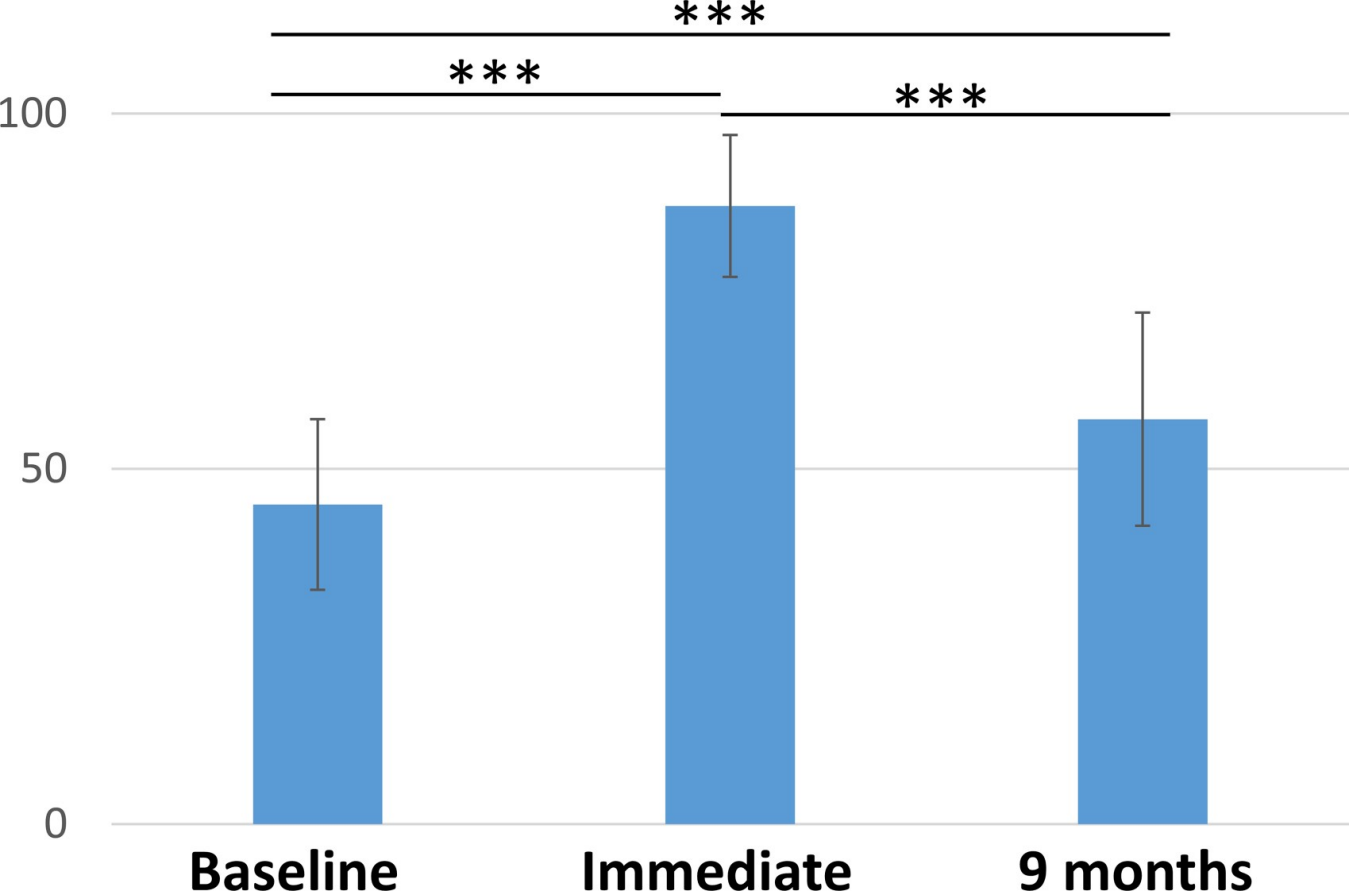
MCQ scores before specific training, immediately after specific training and 9 months later. Scores are expressed out of 100 and the standard deviation is shown. Differences were significant for all comparisons (p < 0.001). MCQ: multiple choice questionnaire.

The 176 students with specific training obtained a mean score of 57 ± 15 for the evaluation of theoretical knowledge. The 20 controls had a mean score of 60 ± 15 (*p* = 0.35; Fig 2). Evolution of the scores for specifically trained students are presented in Fig 3. The 15 emergency medicine residents and fellows had a score of 50 ± 12 (p = 0.12).

### Evaluation of student satisfaction with the specific training

Overall, 93% of the students considered the program to be satisfactory or very satisfactory and to have increased their confidence in their ability to perform LP on patients (7% did not answer this question).

## Discussion

Specific training in LP just before the start of clinical clerkship significantly and durably increased the inclination of medical students to use atraumatic needles. Moreover, the medical students undergoing this training had a level of skill and knowledge after one year of clinical rotation similar to that of students who had completed three years of clinical clerkship without the specific training in LP.

Thus, the main aim of this training course—increasing the use of atraumatic needles—was achieved. The result can look a bit disappointing at first, with only 60% atraumatic needle use after specific training. Nonetheless, the abilities of the 4th-year students were improved to a degree comparable to those of the 6th-year students. It is noteworthy that this result was obtained nine months after the training. As shown for knowledges, immediate evaluation is excellent and scores decrease on longer-term. Such phenomenon is seen in various forms throughout our medical education system. While forgetting is a known and natural physiological phenomenon, our educational systems often do not account for it [7]. Immediate evaluation would have probably shown almost 100% of appropriate needle use. Nonetheless, the real interest is the long-term use. Indeed, many of the specifically trained students have seen non-optimal practices during their clinical rotations and have reproduced these practices during the OSCE rather than practices learned during the specific training. We can wonder if a booster course could further improve these results.

The medical students acquired sufficient knowledge and skill from this specific training to be able to perform LP right from the first clinical rotation. Operator stress has been shown to be significantly related to patient confidence in the operator and the risk of post-dural puncture headache (PDPH) [8]. Simulation-based training increased both the confidence of the students and the proportion of students choosing to use atraumatic needles. This training program would, therefore, probably increase patient safety and comfort, although it would be difficult to observe this improvement directly.

Despite the publication of American Academy of Neurology guidelines recommending the use of atraumatic needles for LP in 2005 [9], the frequency with which such needles are used remains low worldwide [1]. Following the recent publication of a meta-analysis demonstrating that atraumatic needles reduce PDPH, and are cost-effective and no more difficult to use than cutting needles [1], new guidelines have been issued by an international panel [10]. Another advantage of atraumatic needles is that their use reduces the red blood cell count in the CSF [11]. These new publications should help to accelerate a change in practices. It would be possible to force residents and physicians to change their habits, by making them use atraumatic needles [12], but it is likely to be more effective to teach medical students how to use the most appropriate needles right from the start.

The OSCE was used to evaluate skills and attitudes during the performance of LP [13]. OSCE is used for the evaluation of medical students in neurology, but there are no published studies concerning its use to evaluate LP. We performed the entire evaluation in a single sitting, with a maximum time of 20 minutes allowed, to simulate as closely as possible what would be possible during a mini clinical evaluation exercise (mini-CEX) or a direct observation of procedural skills (DOPS). Both these methods are used to evaluate interactions with or interventions in real patients. In this study, it would not be possible to perform such an evaluation on 40 comparable patients. However, one of our objectives was to perform an evaluation as close as possible to a mini-CEX, which is considered to be one of the best forms of evaluation [14]. We chose to use a conventional checklist, although an error-focused checklist to identify incompetence could also be of interest in the present case [15].

This single training course effectively changed practices, but did not lead to the adoption of atraumatic needles by 100% of the students, possibly because many students observed inappropriate practices during their clinical rotations. Given the large number of LPs performed by neurologists [4], these specialists should play a key role in driving changes in practices. Each neurologist can play a part in the collective effort to train medical students and residents, but also more established physicians.

The main limitation of the present study is the control group. Randomizing 4^th^ year medical students into 2 groups, with or without the specific training and evaluating both groups several months later would be better to assess the impact of this training.

## Conclusion

The goal of this specific training course was to encourage medical students to use the most appropriate needles right from the start. Indeed, it is easier to learn correctly in the first place than to learn outdated practices and then have to change during medical residency or even later. This study shows that a single specific training can increase the use of atraumatic needles by medical students lastingly.

## Supporting information

S1 Appendix Standardized checklist to assess medical students performance during the direct observation of procedural skills (DOPS). The French version used is presented together with a proposed English version. This 21-item evaluation chart was used to evaluate both skills and attitudes, resulting in the awarding of an overall mark out of 100.
(DOCX)

S2 Appendix Multiple choice questionnaire (MCQ) to evaluate theoretical knowledge. The French version used is presented together with a proposed English version. This 10-item MCQ resulted in the awarding of an overall mark out of 100.
(DOCX)

S3 Appendix Raw data for DOPS and MCQ of 4^th^ year and 6^th^ year medical students. For MCQ, 2 scoring systems are provided, noted B1 and B2.
(XLSX)
