# Supplementary material for: Specific lumbar puncture training during clinical clerkship durably increases atraumatic needle use
Source: PLoS One. 2019 Jun 10;14(6):e0218004. doi: 10.1371/journal.pone.0218004 (PMC6557568; doi:10.1371/journal.pone.0218004)
Supplement: S1 Appendix — The French version used is presented together with a proposed English version. This 21-item evaluation chart was used to evaluate both skills and attitudes, resulting in the awarding of an overall mark out of 100. (DOCX) [file pone.0218004.s001.docx]

# Annexe 1. Grille d’évaluation pour l’observation directe des compétences pratiques.

|  | Non fait | Fait partiellement | | | Bien fait |
| --- | --- | --- | --- | --- | --- |
| Dire Bonjour | 0 |  |  |  | 1 |
| Se présenter | 0 |  | 1 |  | 2 |
| Expliquer le geste/ Rassurant / Empathique | 0 | 1 | 2 | 3 | 4 |
| Risque de céphalées (syndrome post-PL) | 0 |  | 1 |  | 2 |
| Pas de risque de paraplégie | 0 |  | 1 |  | 2 |
| Vérifier bilan de coagulation | 0 |  |  |  | 4 |
| Glycémie concomitante | 0 |  |  |  | 2 |
| Patch EMLA pour 1h ou injection de xylocaine | 0 |  |  |  | 2 |
| Proposer MEOPA | 0 |  |  |  | 1 |
| Installation confortable | 0 |  | 1 | 2 | 3 |
| Repères anatomiques adaptés | 0 |  | 1 |  | 2 |
| Tenue adaptée |  |  |  |  |  |
| Gants stériles (0 si faute d'asepsie) | 0 |  |  |  | 2 |
| Masque | 0 |  |  |  | 2 |
| Champ stérile (0 si faute d'asepsie) | 0 |  |  |  | 2 |
| Compresses stériles | 0 |  |  |  | 2 |
| Désinfection 5 temps | 0 | 1 | 2 | 3 | 4 |
| Type d'aiguille utilisé | 20G = 0 |  | 22G = 3 |  | 25G = 6 |
| Durée entre passage de la peau et recueil de la première goutte | > 4 min | 2 à 4 min | 1 à 2 min | 30 à 60 sec | <30 sec |
|  | 0 | 2 | 4 | 6 | 8 |
| Qualité technique globale | 0 |  | 1 | 2 | 3 |
| Volume recueilli | < 2 x 10 gouttes |  | > 3 x 10 gouttes |  | ≥ 3 x 20 gouttes |
|  | 0 | 1 | 2 | 3 | 4 |
| Prévention d'accident d'exposition au sang | 0 |  | 1 |  | 2 |
| TOTAL |  |  |  |  | /60 |
| TOTAL CONVERTI (Total / 0,6) |  |  |  |  | /100 |

Annexe 2 : Questionnaire d’évaluation des connaissances théoriques. **Les réponses justes apparaissent en gras**

Sélectionnez les réponses justes :

1 - La moelle épinière :

**A - se termine généralement au niveau L1-L2**

B - se termine généralement au niveau L2-L3

C - se termine généralement au niveau L3-L4

D - se termine généralement au niveau L4-L5

E - se termine généralement au niveau L5-S1

2 – La ligne passant par le sommet des 2 crêtes iliaques est au niveau de :

A – l’épineuse de L1

B - l’épineuse de L2

C - l’épineuse de L3

**D - l’épineuse de L4**

E - l’épineuse de L5

3 – En pratique quotidienne, la ponction lombaire :

**A – Peut être réalisée en position assise**

**B – Peut être réalisée en décubitus latéral**

C – Peut être réalisée en décubitus ventral

D – Est responsable d’une paraplégie dans 10% des cas

E – Est responsable d’une paraplégie dans 1% des cas

4 – Le syndrome post-ponction lombaire :

A – Est réduit par l’augmentation des apports hydriques

B – Est réduit par le respect d’un décubitus dorsal durant 2 heures suivant le geste

C – Est réduit par le respect d’un décubitus dorsal durant 6 heures suivant le geste

D – Est réduit par le respect d’un décubitus dorsal durant 24 heures suivant le geste

**E – Est réduit par l’utilisation d’aiguilles plus fines**

5 – Pour améliorer l’antalgie, un patch d’EMLA peut être utilisé :

A – il doit être posé au moins 5 minutes avant le geste

B – il doit être posé au moins 15 minutes avant le geste

C – il doit être posé au moins 30 minutes avant le geste

**D – il doit être posé au moins 60 minutes avant le geste**

E – quelle que soit la durée d’application, il n’est pas supérieur à un placebo

6 – Avant une ponction lombaire programmée :

**A – il est important d’expliquer clairement le geste au patient pour le rassurer**

B – il est préférable de ne rien expliquer au patient pour ne pas l’inquiéter

**C – un bilan d’hémostase doit être réalisé**

D – un ionogramme sanguin doit être réalisé

E – un bilan hépatique doit être réalisé

7 – L’analyse minimale du LCR doit comprendre :

**A – une analyse biochimique (Chlore, Glucose, Protéines)**

**B – une analyse cytologique**

**C – une analyse bactériologique**

D – une recherche de bandes oligoclonales

E – une PCR virale

8 – En cas de PL traumatique :

**A – le LCR est plus rouge dans le premier tube que dans le dernier**

B – le LCR est plus rouge dans le dernier tube que dans le premier

C – le surnageant est xanthochromique

D – le risque de syndrome post-PL est majoré

**E – le rapport Globules rouges / Globules blancs est le même que dans le sang**

9 – En cas de syndrome post-PL:

**A – le décubitus réduit les céphalées**

B – le décubitus aggrave les céphalées

**C – la caféine réduit la durée du syndrome post-PL**

**D – le blood-patch est le traitement de référence**

**E – il existe un risque d’hématome sous-dural et de thrombophlébite cérébrale**

10 – Quelles sont les affirmations vraies :

**A – une inhalation d’un mélange 50% protoxyde d’azote – 50% oxygène (MEOPA) peut être réalisée pour réduire la douleur et l’anxiété durant le geste**

B – les aiguilles de 20 Gauges biseautées (Jaune) sont à préférer chez la personne âgée pour obtenir un meilleur débit de LCR

**C – les aiguilles de 25 Gauges atraumatiques (Orange) sont à utiliser en priorité chez tous les adultes afin de réduire le risque de syndrome post-PL**

**D – les aiguilles de 22 Gauges biseautées (Noire) ont un biseau qui doit être orienté parallèlement au grand axe des fibres de la dure mère pour limiter le risque de syndrome post PL**

E – En utilisant une aiguille atraumatique de 25 Gauges (Orange), le risque de syndrome post-PL est diminué de près de 50% par rapport à l’utilisation d’une aiguille de 20 Gauges biseautée (Jaune)
