# Supplementary material for: Specific lumbar puncture training during clinical clerkship durably increases atraumatic needle use
Source: PLoS One. 2019 Jun 10;14(6):e0218004. doi: 10.1371/journal.pone.0218004 (PMC6557568; doi:10.1371/journal.pone.0218004)
Supplement: S2 Appendix — The French version used is presented together with a proposed English version. This 10-item MCQ resulted in the awarding of an overall mark out of 100. (DOCX) [file pone.0218004.s002.docx]

# Appendix 1. Standardized checklist to assess medical students performance during the direct observation of procedural skills.

|  | Not done | Partly done | | | Well done |
| --- | --- | --- | --- | --- | --- |
| Say hello | 0 |  |  |  | 1 |
| Introduce her/himself | 0 |  | 1 |  | 2 |
| Explain the procedure / Reassuring | 0 | 1 | 2 | 3 | 4 |
| Explain the risk of post dural puncture headache | 0 |  | 1 |  | 2 |
| No risk of paraplegia | 0 |  | 1 |  | 2 |
| Check hemostasis | 0 |  |  |  | 4 |
| Check concomitant glycemia | 0 |  |  |  | 2 |
| Use EMLA patch or local anesthetics injection | 0 |  |  |  | 2 |
| Propose 50%N2O-50%O2 | 0 |  |  |  | 1 |
| Convenient installation (patient and student) | 0 |  | 1 | 2 | 3 |
| Appropriate anatomical location for puncture | 0 |  | 1 |  | 2 |
| Sterile gloves (0 if not used properly) | 0 |  |  |  | 2 |
| Surgical mask | 0 |  |  |  | 2 |
| Sterile field (0 if not used properly) | 0 |  |  |  | 2 |
| Sterile compresses | 0 |  |  |  | 2 |
| 5 phases disinfection | 0 | 1 | 2 | 3 | 4 |
| Type of needle used | 20G = 0 |  | 22G = 3 |  | 25G = 6 |
| Time between skin puncture and first drop collected | > 4 min | 2 to 4 min | 1 to 2 min | 30 to 60 sec | <30 sec |
|  | 0 | 2 | 4 | 6 | 8 |
| Overall technical quality | 0 |  | 1 | 2 | 3 |
| Volume drawn | < 2 x 10 gouttes |  | > 3 x 10 gouttes |  | ≥ 3 x 20 gouttes |
|  | 0 | 1 | 2 | 3 | 4 |
| Prevention for accidental blood exposure | 0 |  | 1 |  | 2 |
| TOTAL |  |  |  |  | /60 |
| **CONVERTED TOTAL (Total/0.6)** |  |  |  |  | **/100** |

Appendix 2: Multiple choice questionnaire to evaluate theoretical knowledge. **Correct answers are noted in bold characters.**

Select the right answer (one or more possible answers):

1 – Spinal cord:

**A – Generally ends at L1-L2 level**

B - Generally ends at L2-L3 level

C - Generally ends at L3-L4 level

D - Generally ends at L4-L5 level

E - Generally ends at L5-S1 level

2 – The line joining the superior aspect of the iliac crest posteriorly marks the level of the spinous process of:

A –L1

B - L2

C - L3

**D - L4**

E - L5

3 – In daily practice, lumbar puncture:

**A – Can be performed in a sitting position**

**B – Can be performed in lateral decubitus**

C – Can be performed in ventral decubitus

D – Is responsible for paraplegia in 10% of cases

E – Is responsible for paraplegia in 1% of cases

4 – Post-lumbar puncture headache :

A – Is reduced with higher water intake

B – Is reduced using a dorsal decubitus for at least 2 hours after the puncture

C – Is reduced using a dorsal decubitus for at least 6 hours after the puncture

D – Is reduced using a dorsal decubitus for at least 24 hours after the puncture

**E – Is reduced when using finer needles**

5 – To reduce pain, an EMLA patch can be used :

A – It must be placed at least 5 minutes before the puncture

B – It must be placed at least 15 minutes before the puncture

C – It must be placed at least 30 minutes before the puncture

**D – It must be placed at least 60 minutes before the puncture**

E – It is not superior to placebo, whatever the duration

6 – Before a planned lumbar puncture :

**A – It is important to clearly explain the procedure to the patient to reduce anxiety**

B – It is better not to explain the procedure to the patient to avoid anxiety

**C – Hemostasis must be checked on a blood sample**

D – Ionogram must be checked on a blood sample

E – Liver function must be checked on a blood sample

7 – The minimum analysis of the CSF must include:

**A – Biochemical analysis (Chlore, Glucose, Proteins)**

**B – Cytological analysis**

**C – Bacteriological analysis**

D – Oligoclonal bands test

E – Viral PCR

8 – In case of traumatic LP :

**A – CSF is redder in the first sampling tube than in the last**

B – CSF is redded in the last sampling tube than in the first

C – Supernatant presents xanthochromia

D – Post-dural puncture headache is increased

**E – The ratio between red and white blood cell is identical to the ratio in the blood**

9 – In case of post-dural puncture headache (PDPH) after LP:

**A – Decubitus reduces headaches**

B – Decubitus exacerbate headaches

**C – Cafeine reduces PDPH duration**

**D – Blood-patch is the reference treatment**

**E – There is a risk for subdural hematoma and cerebral venous thrombosis**

10 – Which of these propositions are right?

**A – 50% nitrous oxyde / 50% dioxygen inhalation during the procedure can reduce both pain and anxiety**

B –20 Gauges cutting needles (Yellow) should be preferred in older patient to allow higher CSF flow

**C – 25 Gauges atraumatic needles (Orange) should be preferred in all adults to reduce the risk of post dural puncture headache**

**D –22 Gauges cutting needles (Black) have a bevel that must be oriented parallel to the main axis of dura mater fibers to reduce the risk of post dural puncture headache**

E – Using 25 Gauges atraumatic needles (Orange), the risk to present a post dural puncture headache is reduced of almost 50% compared to 20 Gauges cutting needles (Yellow)
